# Extraluminal colonic arteriovenous haemangioma: an unusual cause of chronic lower gastrointestinal bleeding

**DOI:** 10.1308/003588413X13511609955094

**Published:** 2013-03

**Authors:** SK Somasundaram, G Akritidis, S Alagaratnam, TV Luong, OA Ogunbiyi

**Affiliations:** Royal Free London NHS Foundation Trust,UK

**Keywords:** Arteriovenous haemangioma, Gastrointestinal haemangioma, Colonic haemangioma, Arteriovenous malformation, Segmental colitis

## Abstract

Lower gastrointestinal bleeding is a common general surgical presentation in acute and chronic settings. Vascular anomalies account for 2% of such cases and can therefore be missed. We discuss a rare vascular anomaly in one of our patients where the diagnosis was not established for a ten-year period. We describe the subsequent management and a brief review of the literature of this uncommon condition.

## Case history

A 60-year-old woman was seen initially in a general surgical outpatient clinic about 10 years previously with longstanding per-rectal bleeding that had always been intermittent and fresh bright red in colour. She complained of left-sided abdominal pain prior to opening her bowels and had no change in bowel habits, loss of weight or appetite. Her previous medical history included tuberculosis, an appendicectomy and a hysterectomy. Examination during her initial presentation showed second degree haemorrhoids and a rigid sigmoidoscopy to 10cm was reported as normal. She was treated with topical ointments for her haemorrhoids but re-presented with similar complaints five years later and underwent a colonoscopy that showed an inflamed mucosa at 20cm from the anal verge. Biopsies were performed from this abnormal area of suspected segmental colitis. This led to high volume rectal bleeding, necessitating an overnight stay in hospital for observation. The biopsies were reported to be histologically normal.

**Figure 1:**
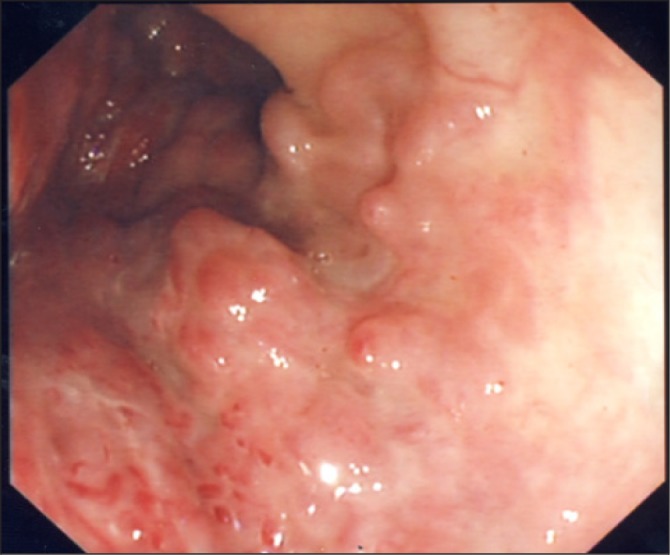
Colonoscopic view of the vascular malformation

**Figure 2:**
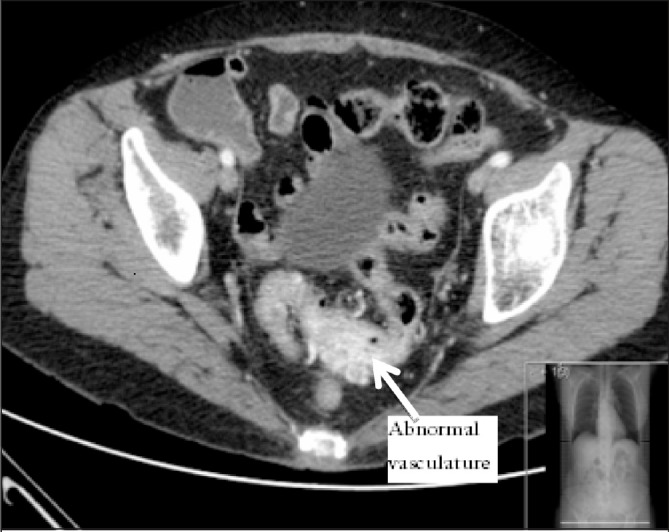
Computed tomography with intravenous contrast; sigmoid thickening demonstrated with abnormal vasculature

**Figure 3:**
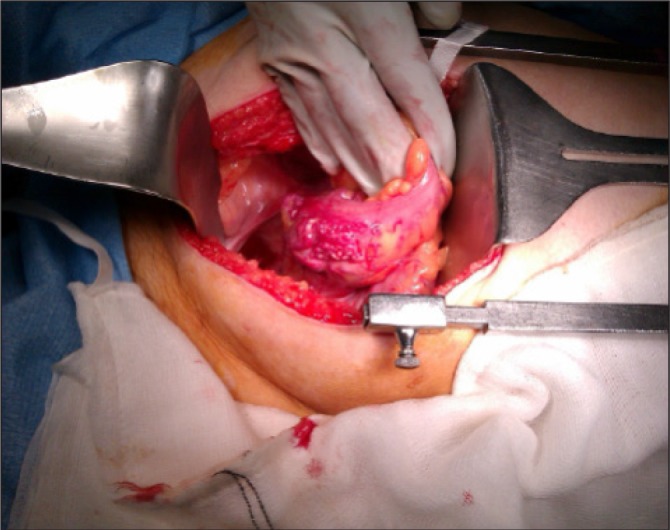
Operative photograph showing the sigmoid colon with extraluminal abnormal vasculature

The patient was re-referred by her general practitioner to our teaching hospital four years later with frequent episodes of large per-rectal bleeding associated with left lower abdominal pain. A flexible sigmoidoscopy booked as a routine investigation demonstrated a bulky and inflamed sigmoid mucosa (Fig 1). The clinical impression was again of segmental colitis or lymphoma. No biopsies were performed owing to the previous history of bleeding, and computed tomography (CT) of her abdomen and pelvis was arranged. This showed a large arteriovenous malformation with abnormal vessels surrounding a 5cm thickened segment of sigmoid colon with large calibre feeding arteries and draining veins (Fig 2). As definitive treatment for her condition, she underwent an open high anterior resection (Fig 3) with an uneventful postoperative recovery.

**Figure 4:**
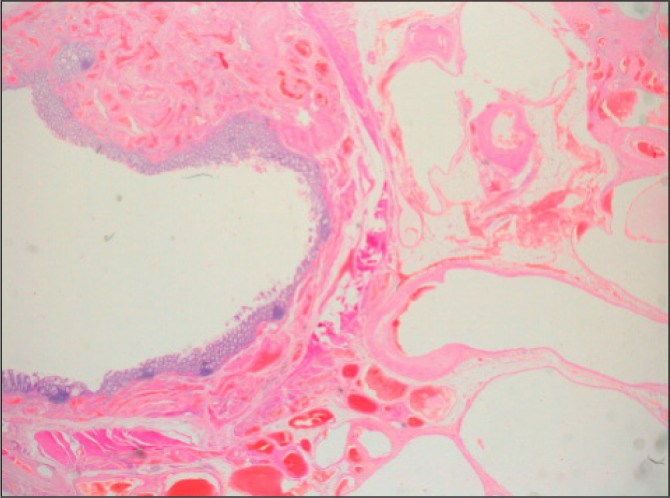
Low magnification histology slide shows extensive involvement of the bowel wall by dilated and deformed vessels, extending from the submucosa through the muscularis propria into the pericolic adipose tissue. The mucosa is intact and shows no evidence of significant pathological abnormalities

**Figure 5:**
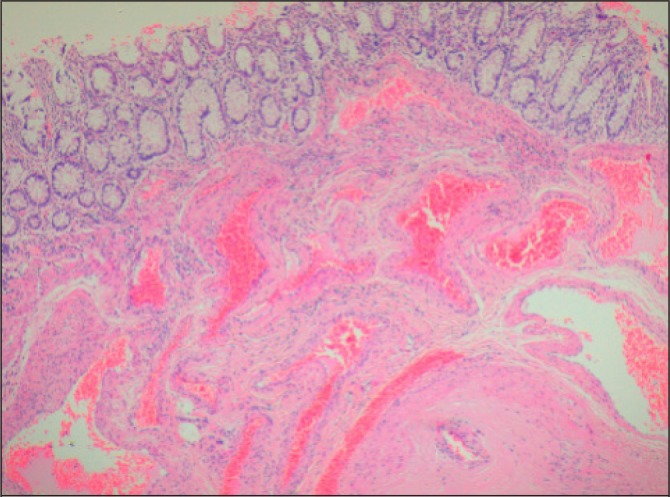
Higher magnification histology slide shows normal colonic mucosa with an underlying submucosal proliferation of thick-walled vessels lined by a single layer of well differentiated endothelial cells

The histology demonstrated an arteriovenous haemangioma, involving the full thickness of the colonic wall from the submucosa, extending widely into the pericolic adipose tissue. The mucosal surface overlying the haemangioma was intact and appeared normal with no evidence of direct involvement or ulceration (Figs 4 and 5).

## Discussion

Our preoperative imaging identified this lesion to be an arteriovenous malformation. Histologically, the lesion was reported to be a haemangioma with arteriovenous components. The terms ‘angiodysplasia’, ‘arteriovenous malformation’ and ‘haemangioma’ have been confusingly used synonymously in the literature. A 2008 review by Regula *et al* suggested that the most logical method of classifying vascular anomalies in the gastrointestinal tract (GI) would be according to anatomic and pathophysiological principles.^1^ Angiodysplasias and angioectasias have been differentiated clearly from the other vascular lesions by the histological appearance of ectatic vascular channels confined to the mucosa and submucosa,^2^ and the clinical tendency for these lesions to be solitary, in the elderly and usually in the right hemicolon.

Cavernous and capillary haemangiomas are by far the most common variants of GI haemangiomas.^3^ They are usually congenital, arising from the submucosal plexus, polypoid and intraluminal. GI arteriovenous malformations are vascular hamartomatous lesions containing arteries and draining veins distributed throughout all layers of the bowel wall.^4^ The likelihood is that the terms ‘arteriovenous haemangiomas’ and ‘arteriovenous malformations’ may have been used synonymously in the literature since arteriovenous haemangiomas appear to be arteriovenous malformations with benign neoplastic changes.

Reports of true colonic arteriovenous haemangiomas are extremely rare and we have only been able to identify two such reports in the literature to date.^3,5^ The endoscopic appearance of our patient’s lesion did not appear like a vascular lesion. In the early stages it mimicked colitis and in later stages it appeared like a neoplastic lesion. Histologically, it spared the mucosa, containing dilated arteries and veins in the rest of the bowel wall. The rarity of the underlying lesion accounts for its late diagnosis and treatment. Repeated biopsies of these lesions could have led to catastrophic bleeding due to the high pressure system but, fortuitously, this stopped spontaneously.

Patients suspected to have these lesions during endoscopy should undergo non-invasive imaging with either contrast enhanced CT of the abdomen and pelvis or magnetic resonance imaging (MRI) of the pelvis. This was evident in our case, where CT revealed the extraluminal vascular abnormality, enabling us to proceed with a definitive management plan. Management of this rare lesion was unlikely to be achieved endoscopically owing to its size and transmural involvement delineated on CT, therefore necessitating surgical resection.

## Conclusions

GI vascular malformations usually present as an acute emergency with massive haematochezia. In an elective setting like in our patient’s case, it required a high index of suspicion to suspect alternative pathologies to account for her longstanding recurrent bleeding. The suspicion was due to the episode of heavy bleeding following her previous biopsies and an abnormal, thickened, inflamed mucosa that was visualised during the flexible sigmoidoscopy. The use of radiological investigations such as CT or MRI should be considered in patients who develop unusual complications such as heavy bleeding following endoscopic biopsies to rule out such lesions non-invasively.
